# Toward an efficient workflow for the analysis of the human milk peptidome

**DOI:** 10.1007/s00216-018-01566-4

**Published:** 2019-02-02

**Authors:** Kelly A. Dingess, Henk W. P. van den Toorn, Marko Mank, Bernd Stahl, Albert J. R. Heck

**Affiliations:** 1 Biomolecular Mass Spectrometry and Proteomics, Bijvoet Center for Biomolecular Research and Utrecht Institute for Pharmaceutical Sciences, University of Utrecht, Padualaan 8, 3584 CH Utrecht, The Netherlands; 2 Netherlands Proteomics Center, Padualaan 8, 3584 CH Utrecht, The Netherlands; 3 Early Life Nutrition, Danone Nutricia Research, Uppsalalaan 12, 3584 CT Utrecht, The Netherlands

**Keywords:** Endogenous peptides, Peptidomics, Biomarker discovery, Human milk, Biofluids

## Abstract

There is a growing interest for investigating endogenous peptides from human biofluids which may provide yet unknown functional benefits or provide an early indication of disease states as potential biomarkers. A major technical bottleneck in the investigation of endogenous peptides from body fluids, e.g., serum, urine, saliva, and milk, is that each of these fluids seems to require unique workflows for peptide extraction and analysis. Thus, protocols optimized for serum cannot be directly translated to milk. One biofluid that is readily available, but which has not been extensively explored, is human milk, whose analysis could contribute to our understanding of the immune development of the newborn infant. Due to the occurrence of highly abundant lipids, proteins, and saccharides, milk peptidomics requires dedicated sample preparation steps. The aim of this study was to develop a time and cost-efficient workflow for the analysis of the human milk peptidome, for which we compared peptide extraction methodologies and peptide fragmentation methods. A method using strong acid protein precipitation and analysis by collision-induced dissociation fragmentation was found to be superior to all other test methods, allowing us qualitative and quantitative detection of about 4000 endogenous human milk peptides in a total analysis time of just 18 h.

**Electronic supplementary material:**

The online version of this article (10.1007/s00216-018-01566-4) contains supplementary material, which is available to authorized users.

## Introduction

The study of endogenous biological peptides from tissue or biofluids was originally termed peptidomics by Schulz-Knappe [1]. Peptidomics has been defined as the comprehensive, qualitative, and quantitative study of all native peptides in a tissue or biofluid [2]. To set a boundary, peptides are mostly defined as low molecular weight compounds (< 10 kDa) [3, 4]. Markedly, these peptides are associated with the proteome of the biofluids from which they originate. These peptides can exhibit distinctly different functions compared to their precursor proteins. They are mostly derived by specific proteases and can be further modified by post-translational modifications (PTMs). Because of their size, endogenous peptides are found throughout the body, in both the intercellular and extracellular spaces, and potentially have unrestricted vascular and interstitial access [3]. Because of these characteristics, the peptidome has become of interest as a diagnostic tool for biomarker discovery for specific disease states [3–7]. Moreover, samples for the analysis of peptidomes from human biofluids, like plasma, saliva, and urine, are readily available and require less invasive sample collections, especially when compared to tissue biopsies.

While endogenous peptides are of biological importance, the analysis of peptidomes is technically less mature compared to studies of cellular or tissue proteomes. The relative immature state of peptidomics can be partly attributed to an overall lack of robust methodological procedures that are able to isolate, measure, identify, and quantify all peptides within a biofluid of interest. Some of the factors attributing to the lack of reliable and repeatable methods are the low concentrations of endogenous peptides, their heterogeneity in size, structures, PTMs, and chemical characteristics [3, 4, 6–8]. Thus, to isolate peptides from complex sample matrixes while remaining MS compatible, specific and efficient extraction protocols are essential. Like the proteome, a typical peptidome covers a high dynamic range, whereby highly abundant peptides potentially suppress the signal of less abundant peptides. LC-MS analysis of natural peptidomes is also more cumbersome than for instance the analysis of a proteome with a tryptic digest. The additional challenges in peptidome analysis originate from factors such as the wider range of size heterogeneity, ranging from 3 to 100 amino acids [4], and that these endogenous peptides do not harbor specific C-terminal residues (R or K for tryptic peptides). These latter residues dictate often the specific charge and fragmentation of tryptic peptides. Not obeying such rules, the analysis of peptides from natural peptidomes requires adjustments in fragmentation schemes and database search strategies employed. Consequently, improvements in peptidomic workflows are needed as there remain pivotal pitfalls throughout the entire workflow.

Here, we aim to improve peptidomic workflows for human milk focusing on sample extraction, MS fragmentation, and database search strategies. Human milk has relatively high concentrations of endogenous peptides [9]; however, they still need to be separated from other abundant milk constituents such as fats, sugars, and proteins. Human milk therefore requires specific protocols, different from common methods used in the analysis of other biofluids, which may include acetonitrile (ACN) and or weak acid precipitation, solid-phase extraction (SPE), liquid–liquid extraction (LLE), size exclusion chromatography (SEC), and filter-aided methods. Instead, for human milk protein precipitation methods, using strong acids are typically favored, which have not been well examined in biofluids such as serum and plasma [10–12].

We first tested extraction methods to optimize for a maximum number of unique peptides identified versus method time. In addition to obtaining higher peptide identification rates by extraction efficiency, different peptide fragmentation methods were explored. These methods included classical and hybrid fragmentation methods, which create a combination of b and y ions and c and z ions, and a decision tree fragmentation method. The software Proteome Discover (PD) 2.2 and SEQUEST HT [13] database search engine were used to analyze the raw data files, evaluating unspecific, semi-tryptic, and tryptic settings in the search algorithms, for peptide identification and label-free quantification (LFQ).

## Materials and methods

### Chemicals and materials

All chemicals used were of HPLC-grade purity. Unless otherwise specified, all chemicals and reagents were obtained from Sigma-Aldrich (Steinheim, Germany). Formic acid (FA) was from Merck (Darmstadt, Germany). Acetonitrile (ACN), chloroform (CHCl_3_), and methanol (MeOH) were purchased from Biosolve (Valkenswaard, The Netherlands). The Sep-Pak C18 1cc vac cartridges were purchased from Waters (MA, USA). MilliQ was produced by an in-house system (Millipore, Billerica, MA).

### Human milk samples

Five human milk samples were used throughout our analysis. One sample was a pooled sample consisting of milk from six different donors. The other four samples were from the same donor over lactation. All samples used were donated to Danone Nutricia Research, by healthy volunteers, in accordance with ethical standards.

### Extraction of endogenous peptides

For all methods, samples were thawed on ice with addition of protease inhibitor. The protease inhibitor was prepared as one tablet of cOmplete Mini EDTA-free (Sigma-Aldrich) in 1 mL of MilliQ water and then added to the samples as one ninth of the total volume. Most samples consisted of volumes of 5 mL. The samples were aliquoted into 1 mL volumes for further analysis.

#### Acid precipitation

The acid precipitation method used was based upon previously described protocols with some modifications [10–12]. A frozen 1 mL aliquot of human milk was thawed on ice. Samples were then assessed as either whole milk or skimmed milk. For whole milk, 20% trichloroacetic acid (TCA) was added to the samples as 1:1 (*v*/*v*), followed by the addition of CHCl_3_ 1:1 (*v*/*v*). The sample was centrifuged at 1500*g*, 4 °C, 10 min. The supernatants were moved to a new tube. Samples were then washed with 100 μL of MilliQ H_2_O + 100 μL of MeOH and then vortexed and centrifuged at 1500*g*, 4 °C, 10 min. Subsequently, the supernatants were collected and combined. For skimmed milk, samples were defatted and skimmed by centrifugation at 1500 g, 4 °C, 10 min. Peptides were isolated from skimmed milk using TCA as 1:1 (*v*/*v*). Working ranges of TCA between 5 and 20% were tested on the skimmed milk samples. For both whole milk and skimmed milk, samples were desalted using Sep-Pak C18 50 mg cartridges (Waters), eluted with 80% ACN/0.1% FA and dried down by speed vac at 45 °C. Samples were stored at − 80 °C until analysis by LC-MS/MS, upon which they were reconstituted in 0.1% FA and then further diluted in 10% FA to achieve an injection of 200–800 ng of material on column.

#### Liquid–liquid extraction

The method was based upon previously described methods with modifications [14–16]. From the same aliquot used for the acid precipitation method, 150 μL of whole milk was mixed with 450 μL of MeOH, vortexed and briefly centrifuged. This was then followed by the addition of 400 μL of CHCl_3_, vortexed and briefly centrifuged, and finally the addition of 200 μL of MilliQ H_2_O. Samples were then extensively mixed on a table top thermo mixer, at room temperature for 10 min at 400 rpm, followed by centrifuging for 1 h at 21,000*g*, 4 °C. For further phase separation, an additional 1 mL of MilliQ H_2_O and 1 mL of MeOH were added and samples were centrifuged for an additional 30 min at 21,000*g*, 4 °C. As mentioned above, samples underwent an SPE cleanup step followed by drying and storage at − 80 °C until analysis by LC-MS/MS, injecting a range of 200–800 ng.

#### Filter aided

From the same aliquot used for the acid precipitation method, 100 μL of whole milk and 100 μL of skimmed milk were used to test filter-aided separation methods. Samples were placed in the tubes with either 5- or 10-kDa filters (Vivaspin^®^ 500) and centrifuged at 15,000*g* for 4 h at 4 °C, per manufacturer’s instructions. For both whole milk and skimmed milk, as mentioned above, samples underwent an SPE cleanup step followed by drying and storage at − 80 °C until analysis by LC-MS/MS, injecting a range of 200–800 ng.

### Mass spectrometry

Endogenous human milk peptides were analyzed by nanoscale LC-MS/MS using an Agilent 1290 Infinity HPLC system (Agilent Technologies, Waldbronn, Germany) coupled on-line to an Orbitrap Fusion Tribrid mass spectrometer (Thermo Fisher Scientific, Bremen, Germany). Reversed-phase separation was accomplished by first trapping peptides using a Reprosil C18 column (3 μm, 2 cm × 100 μm) (Dr. Maisch GmbH, Ammerbuch-Entringen, Germany). The trapping column was coupled to an analytical column, Poroshell EC-C18 (2.7 μm, 50 cm × 75 μm) (Agilent Technologies, Amstelveen, The Netherlands). All columns were made in house. Trapping was performed for 5 min in solvent A, 0.1% FA in water, followed by a gradient of the following: 0–10% solvent B, 0.1% FA in 80% ACN in 10 s, 10–36% in 110 min, 36–100% in 3 min, hold for 1 min, 100–0% in 1 min, and hold for 11 min. Flow was passively split to 300 nL/min.

The mass spectrometer was operated in data-dependent mode (DDA), automatically switching between MS and MS/MS. Full-scan MS spectra from *m*/*z* 375–1500 were acquired at a resolution of 60,000, with and an AGC target of 4.0e5. The most intense precursor ions were selected for fragmentation for a duration of 3 s using 24-s dynamic exclusion. All fragmentation types tested were as follows: collision-induced dissociation (CID), higher-energy collision-induced dissociation (HCD), electron-transfer dissociation (ETD), electron-transfer collision-induced dissociation (ETciD), electron-transfer higher-energy collision-induced dissociation (EThcD), and CID/EThcD. Target peaks were isolated in a 1.6-Da isolation window and subjected to CID, HCD, ETD, ETciD, EThcD, or CID/EThcD in independent runs with normalized collision energy values ranging from 25 to 35% depending on the fragmentation method. See Electronic Supplementary Material (ESM) Table S1 for details on each method. MS/MS spectra were acquired with a resolution of 30,000 using an AGC setting of 1e5 ions with a maximum injection time of 120 ms. Charge state screening was enabled, and precursors with an unknown charge state or a charge state of 1 were excluded. For the decision tree strategy, CID and EThcD fragmentation were performed with normalized collision energies of 35% and 25% respectively. Fragmentation was done based on charge state. CID was selected for charge states of 2–3; and for EThcD, charge states 4–20 were selected. All parameters were the same as those of other fragmentation methods already stated.

### Data analysis

Database searching was performed using Proteome Discover 2.2 (Thermo Scientific) software and SEQUEST HT [13] search engine. Processing nodes included spectrum file reader, Minora feature detector (for LFQ), spectrum selector, SEQUEST HT, and Percolator. SEQUEST HT settings included a complete reviewed human proteome FASTA file from UniProt (2017-07) containing 20,205 proteins. Additional settings included semi-tryptic digest with maximum missed cleavages of 12, a minimum peptide length of 6, and a maximum peptide length of 150. The precursor mass tolerance was set to 10 ppm with a fragment mass tolerance of 0.02 Da, standard search setting for raw data acquired on Orbitrap Fusion Tribrid MS. A preview search with the samples was conducted to identify the most abundant PTMs to be included in our analysis and to identify the greatest number of total peptides. According to this preview search, the most abundant PTMs were oxidation (M), phosphorylation (S, T, Y), and N-terminal acetylation, which therefore were included in the final searches for peptide identifications. No static modification was selected as samples were not reduced and alkylated. Data filtering in Percolator was set to 1% false discovery rate (FDR). A standard consensus workflow was used, with comprehensive and enhanced annotation for LFQ and precursor quantification. Default settings were selected for all nodes with the exception of the precursor ion quantifier node; where, under normalization and scaling, none was selected for both, and under ratio calculation, summed abundance based was selected. A filtering score for SEQUEST Xcorr of 2.0 was applied on the peptide and peptide spectrum match (PSM) level for data analysis. The statistical analysis and all sequence plots were generated with R version 3.4.2, using ggplot2 (version 2.2.1). Gravy scores and theoretical isoelectric points (pIs) were calculated with a script using the ProteinAnalysis class from BioPython 1.5.7. Statistical tests included Pearson correlations with significant *p* values for testing method reproducibility.

## Results and discussion

**Fig. 1:**
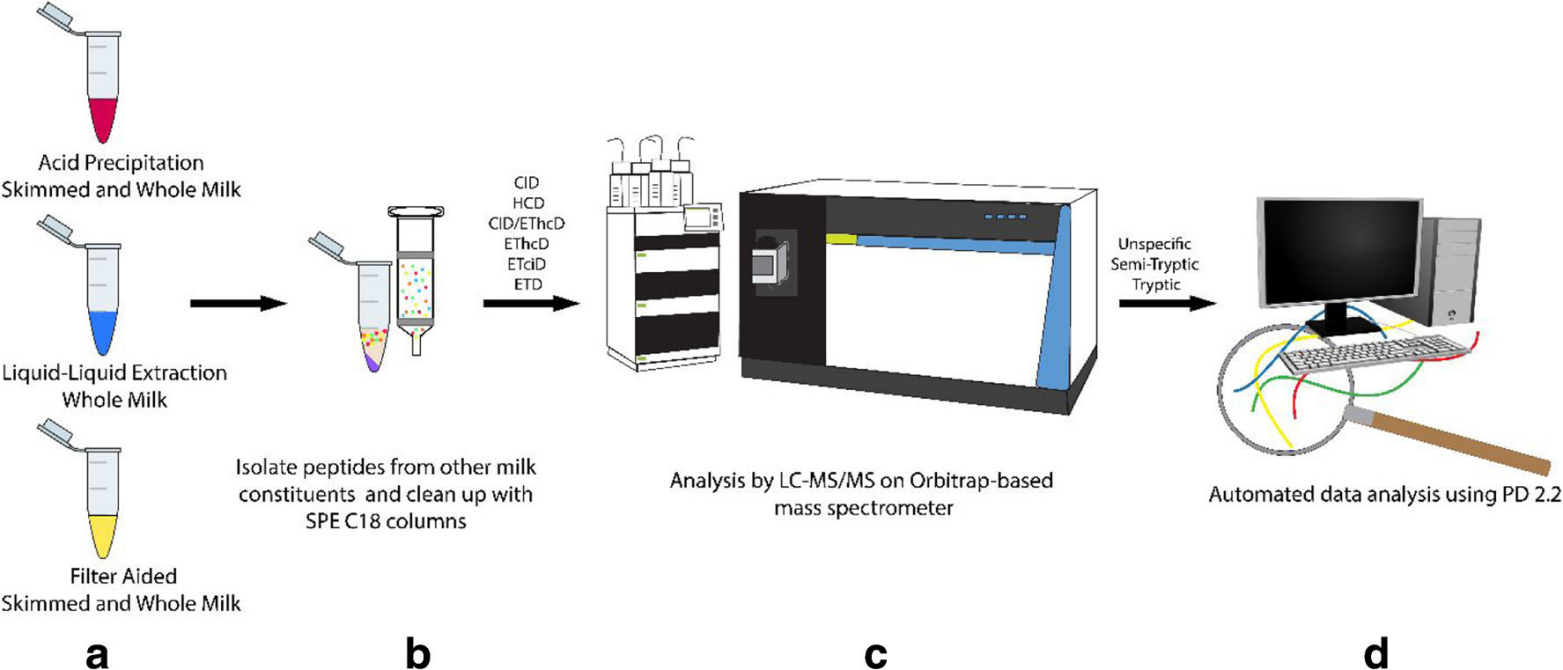
Overview of the modules tested to optimize a workflow for the analysis of the human milk peptidome. Depicted from left to right are the (**a**) extraction methods, (**b**) cleanup methods, (**c**) fragmentation methods, and (**d**) database search methods

Here, we aimed to establish a rapid and cost-effective workflow for the analysis of endogenous peptides from human milk. We argued that an optimized workflow would be the one that enables the identification of a high number of total and unique peptides across a large dynamic range in a relatively short amount of time. Therefore, several extraction methods were compared and rated upon the maximum number of unique peptides identified over the total method time used. In addition, to increase the identification rate of different peptides, MS fragmentation methods were evaluated. Moreover, in the database search, digestion settings were varied to maximize the identification rate while reducing the search time and false-discovery rate (FDR). An overview of the tested variables in the workflow is depicted in Fig. 1.

### Evaluation of extraction methods

The evaluated extraction methods were selected based on prior reported methods used in the literature for biofluids, especially human milk. The most commonly reported method for the analysis of endogenous peptides in human milk is acid precipitation with SPE cleanup prior to LC-MS/MS analysis [10–12]. Though acid precipitation methods are commonly used for other biofluids such as plasma and saliva, these methods tend to use weaker acids at low concentrations (< 1%), such as FA and acetic acid [3, 4, 17]. Alternatively, there are varying protein precipitation methods commonly used in other biofluids, including the use of ice-cold acetone or ACN [3, 4, 17, 18]. Here, we chose to test three different methods for the extraction of endogenous peptides in human milk: an acid protein precipitation method utilizing varying TCA concentrations, an alternative chemical method by LLE with CHCl_3_/MeOH, and a filtration-based method using 5- or 10-kDa cutoff filters.

So far, in the analysis of endogenous peptides from milk, strong acid protein precipitation is commonly used employing TCA at a concentration of 20% [10–12]. We choose to test a broader range of concentrations (5–20%), as the use of high concentrations of TCA for protein precipitation has been hypothesized to cause unwanted peptide aggregation [19]. On the other hand, higher concentrations of TCA have been reported as needed for complete precipitation of β-lactoglobulin from bovine milk [20]. Since human milk also contains heavily glycosylated whey proteins in high concentrations, we hypothesized that higher concentrations of TCA would be needed for complete protein precipitation for efficient peptide isolation. Across the four TCA concentrations tested, 20% provided the highest number of unique peptides identified, 3149, though all tested concentrations were similar in unique peptide yield. The other three concentrations of TCA (5–15%) provided unique peptides of 3123, 3148, and 3042, respectively. Therefore, 20% TCA was used in the subsequent comparison to alternative extraction methods.

The differing methods were evaluated for both whole milk (WM) and skimmed milk (SKM) samples. This is an important parameter to consider in the evaluation of human milk peptidomes as some peptides may be retained in the milk fat globule membrane, and thus missed in SKM samples. All methods included an SPE C18 cleanup step prior to LC-MS/MS analysis. All samples were independently analyzed and measured in duplicate across seven different testing parameters.

**Fig. 2:**
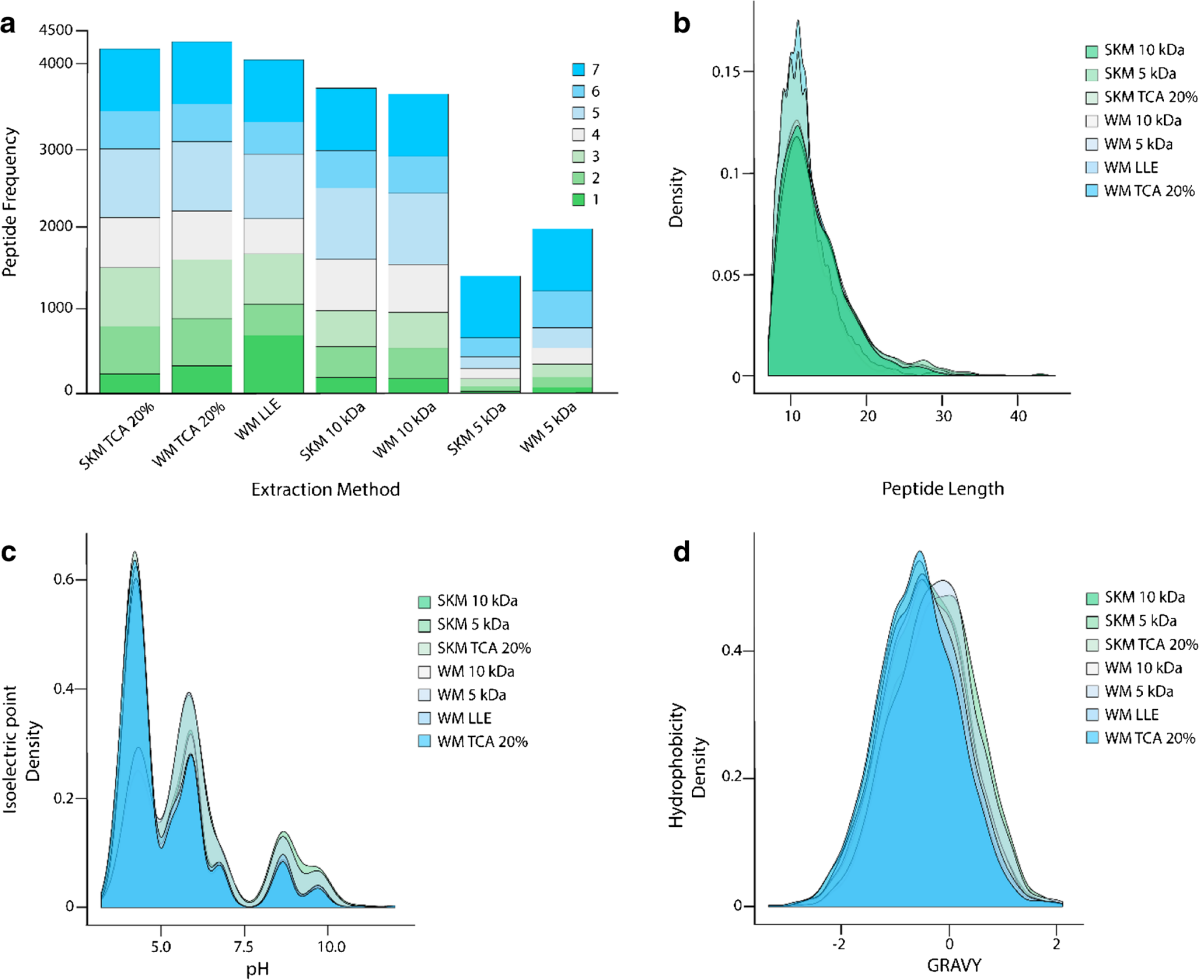
Peptide counts and characteristics for each tested extraction method. (**a**) Stacked bar chart wherein the seven different extraction methods are summarized by peptide counts. Each bin in the stacked bar chart represents how many times a peptide was identified across the seven differing extraction methods, where 1 means a peptide was exclusively identified in only that extraction method and 7 means the peptide was identified across all extraction methods. Physical–chemical properties of the peptides per tested method: peptide length (**b**), isoelectric point (**c**), and GRAVY score (**d**). ESM Table S2 provides numeric information for each bin across the 7 extraction methods in this figure

The seven evaluated extraction parameters in our human milk peptidome analysis included SKM and WM with TCA (20%) protein precipitation, WM by using LLE with CHCl_3_ and MEOH, and SKM and WM by using either a 5- or 10-kDa cutoff filter. Results from this analysis are depicted in Fig. 2 and Table 1. From this analysis, we could determine that the SKM and WM TCA (20%) extraction methods provided the highest absolute number of unique peptides, 3149 and 3237 respectively. These values equated to identification rates of 20 and 21% respectively, while all other extraction methods resulted in identification rates below 20%. Identification rates were determined as a percentage of peptides divided by PSMs. All WM methods provided a greater number of unique peptides than their SKM counter method, most likely due to co-isolation of peptides from within the milk fat globule membrane. The only exception to this was with the 10-kDa filtration method, where the SKM sample resulted in more unique peptide identifications than the WM sample. This could be due to clogging of the filter membrane by fat from the WM sample. Using WM LLE, we identified 713 exclusive peptides that were not identified across the other six extraction methods. The SKM and WM filtration methods using the 10-kDa filter were comparable to the acid precipitation and LLE methods, with 2904 and 2876 unique peptides identified respectively. Both the SKM and WM filtration methods using the 5-kDa filter appeared to be sub-optimal for peptide isolation compared to every other method, with only 1026 and 1557 unique peptides identified, respectively.

**Table 1:** Comparison of extraction, fragmentation, and database searching workflows and total analysis times

Extraction method	Unique peptides	Total peptides	Protein groups	PSMs	MS/MS events	ID rate (%)	Extraction time (hours)	Strengths	Weaknesses
SKM TCA, 20%	3149	4261	796	10,034	51,115	20	3	Easy/fast	May miss some lipid membrane-bound peptides
WM TCA, 20%	3237	4337	815	10,536	50,507	21	4	Easy/fast	Use of hazardous chemicals (chloroform)
WM LLE	3167	4047	754	8782	51,928	17	8	High, unique peptide yield	Labor-intensive/many steps
SKM, 10 kDa	2904	3776	766	8071	50,300	16	4	Easy/fast	Potential loss of peptides due to filter clogging
WM, 10 kDa	2876	3702	733	8271	50,463	16	4	Easy/fast	Potential loss of peptides due to filter clogging
SKM, 5 kDa	1026	1453	345	2789	42,488	7	4	Easy/fast	Potential loss of peptides due to filter clogging/low peptide recovery
WM, 5 kDa	1557	2034	462	4161	47,724	9	4	Easy/fast	Potential loss of peptides due to filter clogging/low peptide recovery
Fragmentation method	Unique peptides	Total peptides	Protein groups	PSMs	MSMS events	ID rate (%)	Analysis time (hours)		
CID	2839	3988	679	10,556	53,975	20	3		
HCD	2813	3870	718	10,414	59,112	18	3		
CID/EThcD	2742	3819	652	10,511	52,590	20	3		
EThcD	1857	2521	371	6653	24,381	27	3		
ETciD	1695	2464	349	5848	23,729	25	3		
ETD	562	973	188	1560	24,528	6	3		
Digestion method									
Unspecific	3932	4724	817	10,802	51,115	21	52		
Semi-tryptic	3310	4016	815	8968	51,115	18	12		
Tryptic	1789	2131	789	4439	51,115	9	2		

While the WM LLE method appears advantageous with the identification of more than 700 exclusive peptides, a major limitation of this method is the time needed and the lower number of unique peptides identified compared to the strong acid protein precipitation methods. The LLE method takes approximately 8 h, compared to 3–4 h for the TCA protein precipitation methods. Figure 2a depicts a comparison of the seven tested methods and how often a peptide was identified across each method, with one being exclusively identified in only that method and seven being identified across all methods. From this data, we conclude that the WM LLE method provided more exclusive peptides than any other method; however, this did not translate to an overall high number of unique peptides identified. Therefore, the cost of time with this method did not outweigh the benefit.

We next evaluated the physical and chemical properties of the identified peptides across all tested methods to examine whether there were specific biases across the methods before determining an optimal method. The physical-chemical properties assessed across all identified peptides included the peptide length, pI, and the grand average of hydropathy (GRAVY) score. The results of these evaluations are depicted in Fig. 2b–d. From this, we could determine that there were no major biases between extraction methods. This was not overall surprising, since the identified peptides across all methods followed similar trends where more than 70% of the peptides were derived by the precursor proteins β-casein, osteopotin, polymeric immunoglobulin receptor, α-S1-casein, and κ-casein. Additionally, these results are in line with those of previous publications [9, 10, 12, 21, 22], where the greatest number of peptides identified were derived from the same five precursor proteins.

We concluded that the TCA (20%) protein precipitation method was the best extraction method for the evaluation of human milk peptides from SKM and WM samples. The extraction of peptides from SKM samples has the advantage of time whereas the extraction of WM samples provides a more comprehensive analysis of endogenous peptides. The TCA method used during our method development analysis was based on the TCA method used by Dallas et al. [10]. This group provided further support for this method by determining that peptides isolated with TCA were not the result of continued proteolytic digestion during sample preparation. This was determined by comparing frozen samples to fresh samples that were treated with boiling for 5 min, to deactivate any protease activity. Results from this study indicated that there were no differences in peptide identification between the frozen and fresh deactivated milk samples, with 96% of all unique peptides overlapping [10]. It can be concluded then that proteolytic activity during sample preparation is not responsible for the identified peptides in this method.

### Evaluation of MS/MS fragmentation methods

**Fig. 3:**
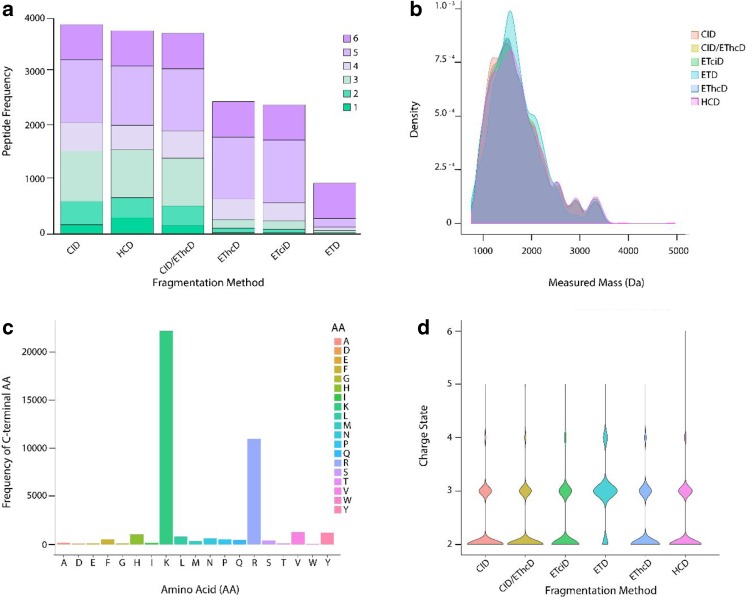
Peptide counts and characteristics for each tested fragmentation method. (**a**) Stacked bar wherein the six different fragmentation methods are summarized by peptide counts. Each bin in the stacked bar chart represents how many times a peptide was identified across the differing fragmentation methods. Physical–chemical properties of the peptides per tested fragmentation method: (**b**) distribution of peptide mass (Da). (**c**) C-terminal AA frequency (in total for all fragmentation methods). (**d**) Distribution of charge states of identified peptides. ESM Table S3 provides numeric information for each bin across the six fragmentation methods in this figure

Although some studies investigating peptidomes of biofluids have explored alternative fragmentation methods [6, 8], they have, as far as we are aware, not yet been explored in the analysis of the human milk peptidome [23–26]. We evaluated the different fragmentation methods using the above-described TCA 20% extraction method with SKM samples, as this gave us the best results for peptide extraction in the shortest amount of time. For our analysis, each sample was independently analyzed and tested in duplicate for each of the six applied fragmentation methods. In these 12 LC-MS runs, 3988, 3870, 3819, 2521, 2464, and 973 peptides (including redundant identifications) were identified by CID, HCD, CID/EThcD, EThcD, ETciD, and ETD, respectively (Fig. 3a and Table 1). Unique peptides followed this same trend. In the number of PSMs identified, CID fragmentation outperformed all other methods, providing the highest total number of peptides and most unique peptide identifications (Table 1). However, HCD fragmentation identified peptides from more protein groups than any other fragmentation method (Table 1).

Upon further analysis of this data, we observed that the majority of our peptides appears to be tryptic like, with a mean measured mass of around 1500 Da and a high majority of peptides with C-terminal residues ending in lysine/arginine cleavages (Fig. 3b and c, respectively). Additionally, the mean peptide length of our samples was 12–13 AAs with the majority of peptides exhibiting, following ESI ionization, a + 2–3 charge state, with only the ETD method having a higher mean charge state of + 3–4 (Fig. 3d). Because of this observed tryptic-like nature of the peptides in the milk peptidome, it would be expected that CID/HCD performed best.

Recently, novel fragmentation methods such as ETD and EThcD have been used to assess endogenous peptides due to the advantage of ETD-based fragmentation in improved sequencing of larger peptides. A study by Sasaki et al. [8] used CID and ETD to profile peptides from secretory granules of a human endocrine cell line. They determined that, even though CID fragmentation identified more peptides, ETD provided an advantage in identifying larger peptides that had antimicrobial functionality. Additionally, other studies have shown that EThcD was advantageous for assessing other endogenous, non-tryptic peptides such as glycosylated human leukocyte antigen (HLA) class I-bound peptides [27]. These peptides, identified due to EThcD fragmentation, would have otherwise not been identified with more classical fragmentation methods such as CID or HCD alone. Additionally, this study showed that HCD fragmentation with collision energy of 35, higher than the recommended setting of 30 for bottom–up proteomics studies, was advantageous in the analysis of endogenous peptides. It was due to this analysis that we choose to use a collision energy of 35 with HCD fragmentation in our analysis.

**Fig. 4:**
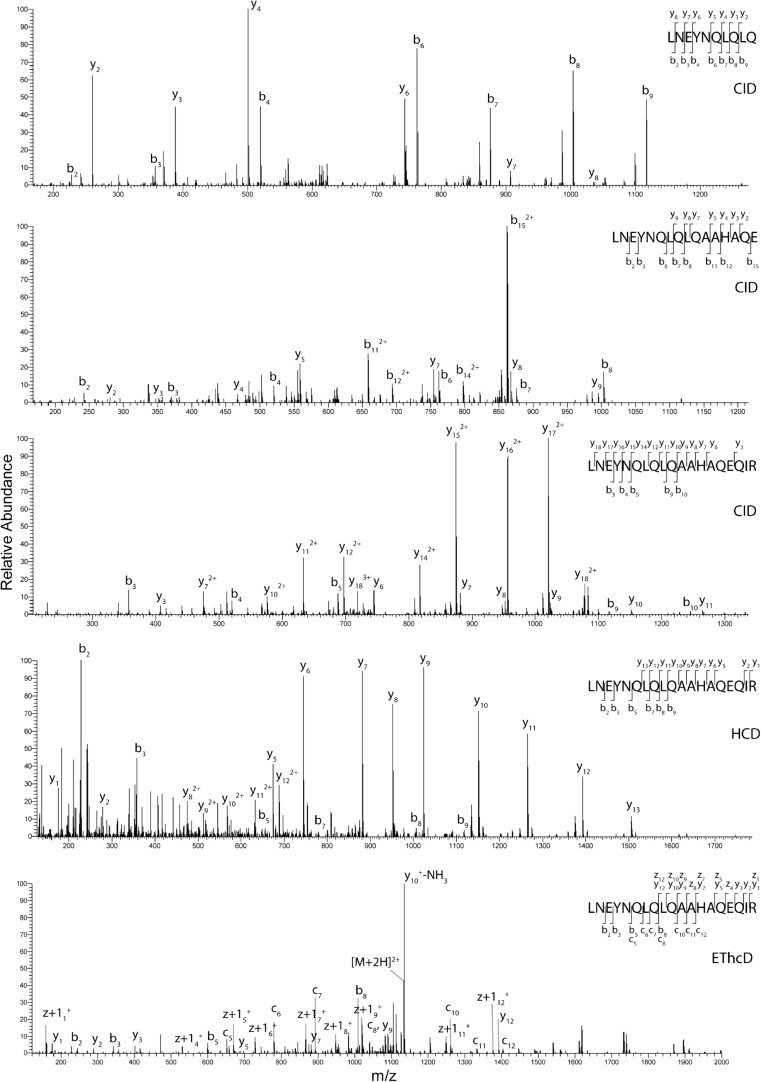
CID fragmentation spectra of the endogenous ladder peptide series from the protein α-S1-casein. The ladder peptide series begins with the shortest peptide LNEYNQLQLQ, followed by LNEYNQLQLQAAHAQE and ending with the longest peptide in the series, LNEYNQLQLQAAHAQEQIR. The longest peptide in the ladder series is also shown with HCD and EThcD fragmentation. The observed fragment ions are indicated in the sequence given in the inset. All three fragmentation methods provide almost full-sequence indicative fragment ions across the ascending ladder peptide series

A common attribute of endogenous peptides are the so-called ladder and/or nested peptides. We observe both of these in our dataset. The endogenous ladder peptide series from the protein α-S1-casein was detected with high-quality spectra and nearly full-fragment ion coverage across all peptides in the series with CID fragmentation (Fig. 4). The ladder peptide series begins with the shortest 10 AA peptide LNEYNQLQLQ, and is extended by several AAs at its C-terminus up to its longest 18 AA peptide in the series, LNEYNQLQLQAAHAQEQIR. We observed in total nine peptides within this series (see ESM Fig. S1). The MS/MS spectrum of the longest peptide in this series is shown with CID, HCD, and EThcD fragmentation in Fig. 4. All three fragmentation methods provided indicative fragment ions across almost the entirety of the peptide backbone for the longest peptide in ladder series.

The application of these fragmentation methods in combination with CID and/or HCD can be implemented in future analysis of the human milk peptidome to provide novel identifications of potentially functional peptides. Aims to address functionality ultimately are impeded by the current ability to identify these peptides, which is limited to matching sequences to those with known functionality or predictive analysis based on sequence structure and functional motifs. Recently, one group did compile a bioactive database which is based on these aforementioned resources and is specific to milk-derived peptides [28]. While this is a good start, more work is needed in this area of research. Implementing novel fragmentation methods complemented with higher resolution scans in the Orbitrap can lead to an improved identification of peptides, especially those carrying more charges. However, to make use of these higher quality spectra, additionally there is a need to optimize database searching strategies for endogenous peptides.

### Evaluation of data analysis strategies

**Fig. 5:**
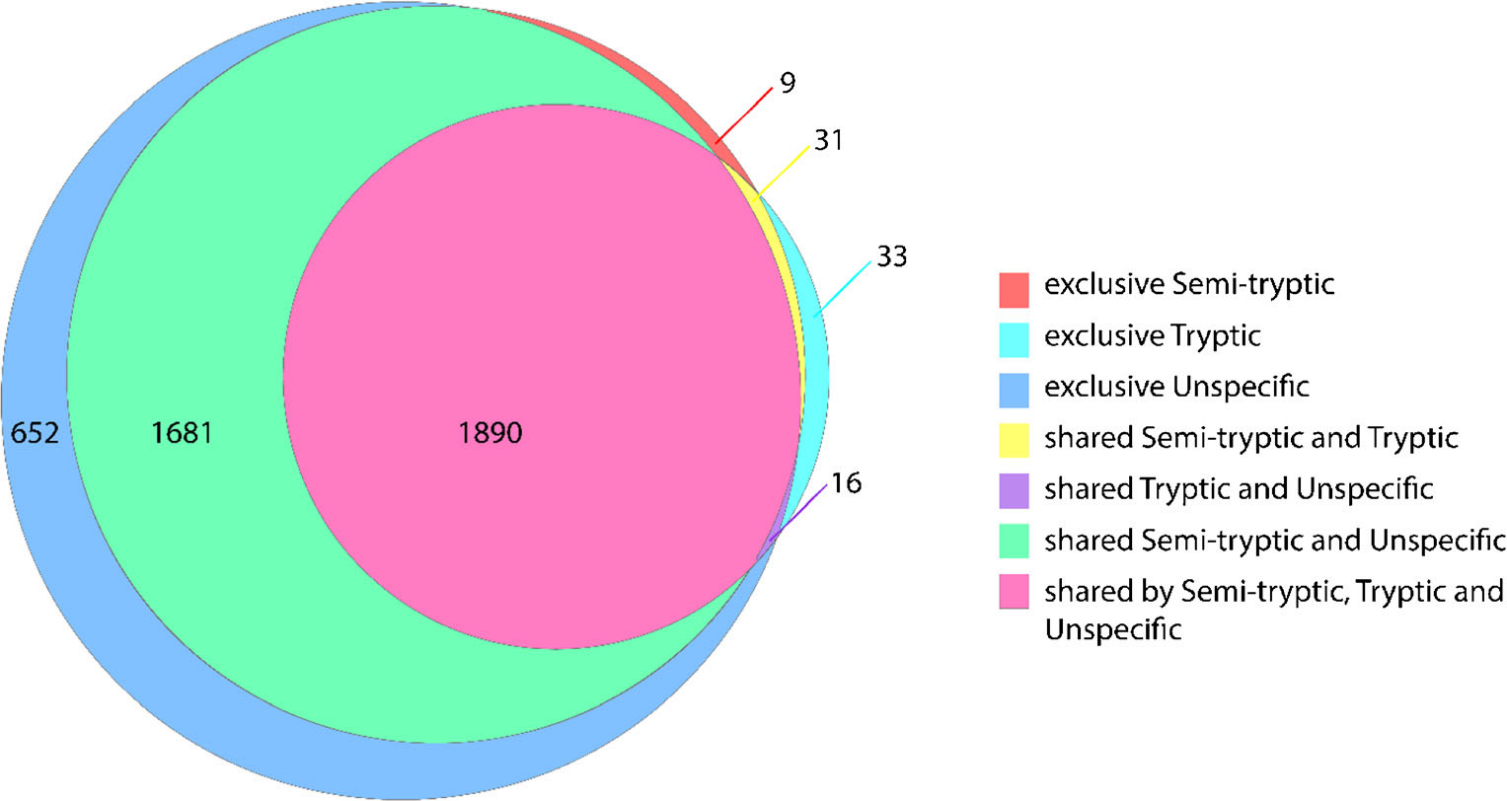
Euler diagram of peptide identifications by using different database search algorithms. The optimized TCA 20% + CID fragmentation method was used. The different settings tested for peptide identifications were tryptic, semi-tryptic, or unspecific. Cumulatively using an extraction method with SKM 20% TCA, CID fragmentation, and semi-tryptic search, we detected in human milk 3310 unique peptides

We next choose to evaluate database searching strategies of our human milk peptides using differing settings in PD 2.2. We choose PD 2.2 as it additionally offers relatively fast and reliable LFQ analysis. The analysis of endogenous peptides typically includes unspecific enzymatic or “no enzyme” searches for identification of peptides by database searching [7]. We investigated the application of searches with semi-tryptic and tryptic digests in comparison to a “no enzyme” search. This choice was made due to the observed nature of the identified peptides, which by large seem to be “tryptic like.” Additionally, it is well supported in the literature that a major enzyme active in human milk is plasmin, which cleaves lysine and arginine (K/R) [7, 10, 21, 29]. As this enzyme is hypothesized to still be active in the mammary gland, it could contribute to the overall semi-tryptic nature of the endogenous peptides we observed in human milk [7, 10, 21, 29]. From this analysis, we observed that semi-tryptic searches provided greater than 85% overlap with the unspecific search. The fully tryptic search only matched the unspecific search with a 46% overlap (Fig. 5). While the unspecific search provided the highest number of total and unique peptide identifications, 4724 and 3932 respectively, this search took approximately 52 h for one sample, while the semi-tryptic search took 12 h (Table 1). Therefore, for an efficient analysis of the most abundant human milk peptides, a semi-tryptic search seems to be the most advantageous.

### Evaluation of reproducibility

Next, we evaluated the qualitative and LFQ reproducibility of our workflow to evaluate whether accurate determination of endogenous peptide abundances can be achieved across a broad range of samples. To assess this, we choose the most optimal workflow, which we determined to be extraction by protein precipitation with TCA 20%, CID fragmentation, and using a semi-tryptic setting in the database searches. To evaluate reproducibility, we choose to examine three different levels, on extraction efficiency with extraction replicates, on MS fragmentation by technical replicates of each extraction replicate and over a range of different sample types with a sample from one donor across different points in lactation.

**Fig. 6:**
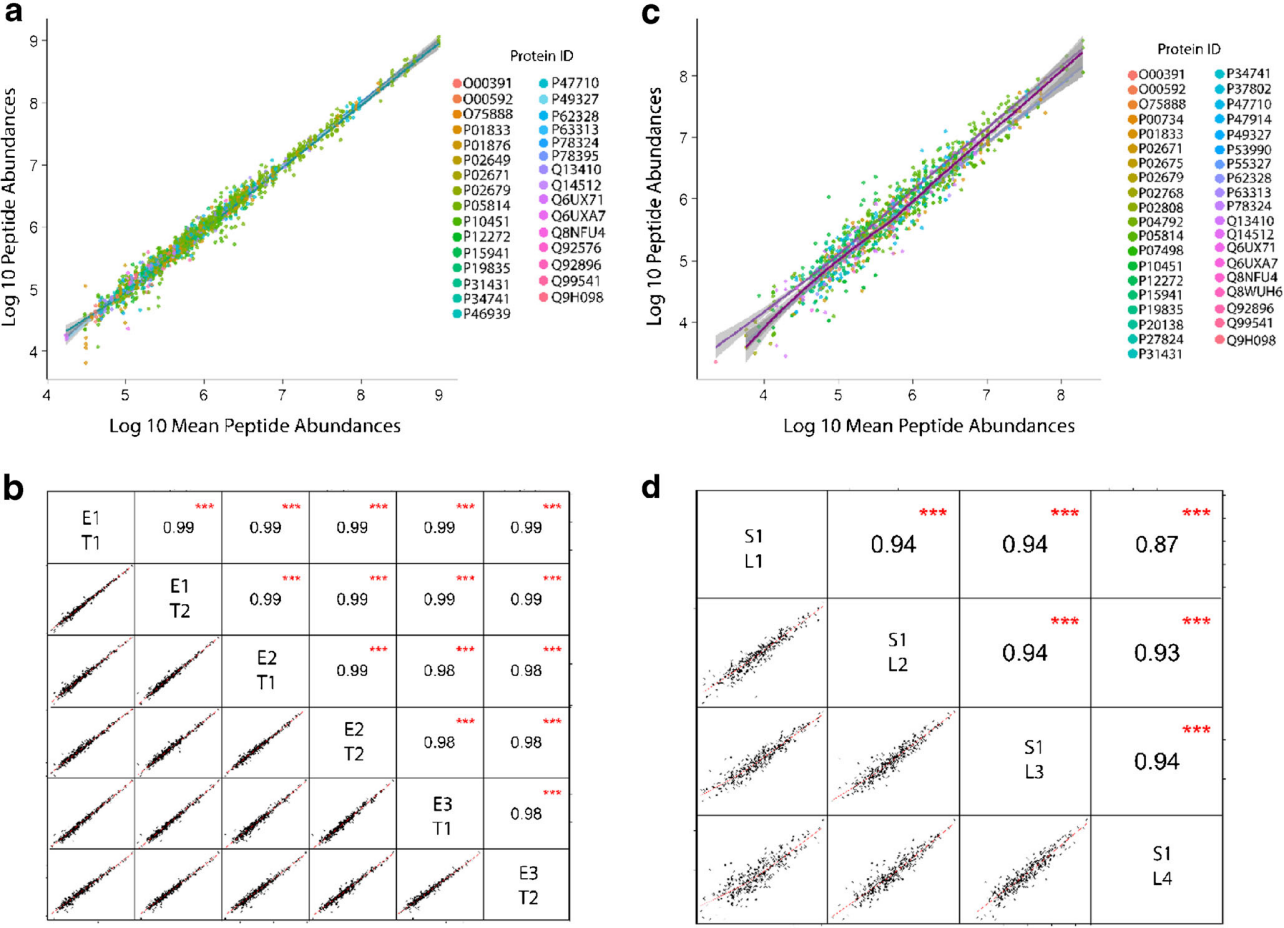
Qualitative and quantitative reproducibility of extraction methods, in both technical and biological replicates. Peptides are colored according to the proteins they originate from. (**a**) The peptide abundance (log10) observed across three extraction replicates, each with two technical replicates, is plotted against the mean peptide abundance. Extraction replicates are designated as E1–3 and technical replicates are designated as T1–2. (**b**) The distribution of each extraction and technical replicate is shown on the diagonal and the x and y axes are the same log proportions as in **a**. On the bottom of the diagonal, the bivariate scatter plots with fitted lines are displayed. On the top of the diagonal, the Pearson correlation and significance level are indicated as stars; three stars indicate a significance level of *p* < 0.001 (**c** and **d**). The same metrics as assessed with milk samples of the same donor across lactation, indicated as S1L1–4 where L1 indicates the earliest lactational stage and L4 indicates the latest

The extraction and technical replicates of one sample are represented in Fig. 6a as the measured peptide abundance of three extraction replicates each with two technical replicates plotted against the mean peptide abundances. The distribution of each extraction and technical replicate is shown as scatter plots with Pearson correlations and significance levels in Fig. 6b. All extraction and technical replicates were highly correlated with Pearson correlations of > 0.98 and a significance level of *p* < 0.001 for all replicates. The same metrics were assessed for differing sample types and are represented in Fig. 6c and d. Varying samples from one donor across lactation were chosen as it is well established that human milk has a changing protein profile across time, with markedly high protein expression within the first few days after birth and then a steady decline over the first year of lactation [30, 31]. Therefore, to show a broad range of the milk peptidome, we thought looking over time would show greater robustness of the method than looking from several individuals all at one time point. The samples across lactation showed as expected a lower, albeit still good, Pearson correlation > 0.87 and a significance level of *p* < 0.001 for all samples. Moreover, all samples and replicates displayed the broad dynamic range of the human milk peptidome, with peptide abundances spanning more than five orders of magnitude. This also shows the robustness of our optimized method to accurately and reproducibly isolate, fragment, and identify endogenous human milk peptides.

## Conclusions

Peptidomics, entailing the comprehensive characterization of all endogenous peptides in a biological sample, is an important sub-discipline of proteomics, which has the potential to become a novel biological explorative avenue for diagnostics and biomarker discovery. Although peptidomics builds upon technologies being introduced for proteomics, the nature and origin of the endogenous peptides analyzed by petpidomics require several different and more specialized approaches. Here, through our analysis of differing extraction, fragmentation, and digestion methods for database searching, we determined the most optimal method for the analysis of endogenous human milk peptides. In summary, this entailed the following: extraction by protein precipitation using 20% TCA, LC MS/MS analysis by CID fragmentation, and database searching using a semi-tryptic setting in the searches. This method gave the overall greatest total number of unique peptides identified in the shortest amount of time in combination with the overall ease of the workflow (see Table 1). Additionally, this workflow was found to be highly reproducible for extraction and technical replicates and for samples of varying lactational stages, e.g., differing protein concentrations. We expect that the work presented here can be used to assist others in making informed decisions about the proper workflow to consider for their own analysis. By optimizing for the highest detection efficiency and shortest analysis time, we expect this workflow will allow us and others to now start to assess tens to hundreds of human milk samples, thereby querying how the milk peptidome varies for instance between healthy and compromised individuals, mothers with differing dietary intakes, and also across lactational stages. Such information may be used to learn more about the relationship between the mother’s milk and the buildup of the infant’s immune system and potentially also be used to optimize the dietary fortifications for infants, such as human milk and or infant formula fortifiers.

## Electronic supplementary material

ESM 1 (PDF 362 kb)

## Abbreviations

AA: Amino acids
ACN: Acetonitrile
CID: Collision-induced dissociation
ETciD: ETD with supplemental CID
ETD: Electron transfer dissociation
EThcD: ETD with supplemental HCD
FA: Formic acid
FDR: False discovery rate
HCD: High-energy collision dissociation
LLE: Liquid–liquid extraction
MS: Mass spectrometry
PD: Proteome Discover
PTMs: Post-translational modifications
SEC: Size exclusion chromatography
SPE: Solid-phase extraction
TCA: Trichloroacetic acid
