# Supplementary material for: Toward an efficient workflow for the analysis of the human milk peptidome
Source: Anal Bioanal Chem. 2019 Feb 2;411(7):1351–63. doi: 10.1007/s00216-018-01566-4 (PMC6449315; doi:10.1007/s00216-018-01566-4)
Supplement: Supplementary file 1 — (PDF 362 kb) [file 216_2018_1566_MOESM1_ESM.pdf]

Analytical and Bioanalytical Chemistry

Electronic Supplementary Material

Toward an efficient workflow for the analysis of the human milk peptidome

Kelly A. Dingess, Henk W. P. van den Toorn, Marko Mank, Bernd Stahl , Albert J. R. Heck

**LNEYNQLQLQ**
**LNEYNQLQLQAA**
**LNEYNQLQLQAAH**
**LNEYNQLQLQAAHA**
**LNEYNQLQLQAAHAQ**
**LNEYNQLQLQAAHAQE**
**LNEYNQLQLQAAHAQEQ**
**LNEYNQLQLQAAHAQEQI**
**LNEYNQLQLQAAHAQEQIR**

Fig. S1 Ladder peptide series as observed from the protein α-S1-casein

Table S1 Description of the MS parameters and the differing MS/MS parameters by fragmentation method

**MS**				
**RF Lens (%)**	**Gradient Time (minutes)**	**AGC target**	**Max IT (ms)**	**Orbitrap resolution**
60	175	4.0e5	50	60000
**MSMS**				
**Fragmentation**	**Collision Energy**	**AGC target**	**Max IT (ms)**	**Orbitrap resolution**
**CID**	35	1.0e5	120	30000
**HCD**	35	1.0e5	120	30000
**ETD**	-	1.0e5	105	30000
**EThcD**	20	1.0e5	105	30000
**ETciD**	35	1.0e5	105	30000
**CID/EThcD**	35 / 25	1.0e5	120	30000

Table S2 Numerical representation of Figure 2A, total peptide count across the seven extraction methods

	SKM TCA (20%)	WM TCA (20%)	WM LLE	SKM 10 kDa	WM 10 kDa	SKM 5 kDa	WM 5 kDa
Exclusive	236	338	713	193	178	23	76
Found in 2 of 7 methods	589	586	389	380	377	63	120
Found in 3 of 7 methods	730	729	620	449	443	91	163
Found in 4 of 7 methods	619	600	438	634	597	130	198
Found in 5 of 7 methods	850	860	802	882	878	145	253
Found in 6 of 7 methods	466	467	393	467	458	230	453
Found in All methods	771	771	771	771	771	771	771
Total	**4261**	**4351**	**4126**	**3776**	**3702**	**1453**	**2034**

Table S3 Numerical representation of Figure 3A, total peptide count across the six fragmentation methods

	CID	HCD	CID/EThcD	EThcD	ETciD	ETD
Exclusive	183	315	168	33	29	13
Found in 2 of 6 methods	446	382	372	86	67	21
Found in 3 of 6 methods	953	910	909	157	158	39
Found in 4 of 6 methods	540	466	518	395	349	68
Found in 5 of 6 methods	1193	1124	1179	1177	1188	159
Found in All methods	673	673	673	673	673	673
Total	**3988**	**3870**	**3819**	**2521**	**2464**	**973**
